# Erratum to: Game-theoretical mapping of fundamental brain functions based on lesion deficits in acute stroke

**DOI:** 10.1093/braincomms/fcab270

**Published:** 2021-11-28

Caroline Malherbe, Bastian Cheng, Alina Königsberg, Tae-Hee Cho, Martin Ebinger, Matthias Endres, Jochen B. Fiebach, Jens Fiehler, Ivana Galinovic, Josep Puig, Vincent Thijs, Robin Lemmens, Keith W. Muir, Norbert Nighoghossian, Salvador Pedraza, Claus Z. Simonsen, Anke Wouters, Christian Gerloff, Claus C. Hilgetag, and Götz Thomalla; Game-theoretical mapping of fundamental brain functions based on lesion deficits in acute stroke. *Brain Communications* 2021. doi: https://doi.org/10.1093/braincomms/fcab204.

In the originally published version of this manuscript, the second paragraph of the abstract was missing from the PDF. The paragraph is as follows:

“Our approach demonstrates a practically feasible strategy for applying an objective lesion inference method to a high-resolution map of the human brain and distilling a small, characteristic set of grey and white matter structures contributing to fundamental brain functions. In addition, we present novel findings of synergistic interactions between brain regions that provide insight into the functional organization of brain networks.”

This error has been corrected online.

